# Curcumin for the clinical treatment of inflammatory bowel diseases: a systematic review and meta-analysis of placebo-controlled randomized clinical trials

**DOI:** 10.3389/fnut.2025.1494351

**Published:** 2025-03-24

**Authors:** Saeid Mohseni, Ali Tavakoli, Hamid Ghazipoor, Neda Pouralimohamadi, Roghayeh Zare, Thomas Rampp, Maryam Shayesteh, Mehdi Pasalar

**Affiliations:** ^1^Department of Persian Medicine, School of Persian Medicine, Babol University of Medical Sciences, Babol, Iran; ^2^Research Center for Traditional Medicine and History of Medicine, Shiraz University of Medical Sciences, Shiraz, Iran; ^3^Department of Family Medicine, School of Medicine, Shiraz University of Medical Sciences, Shiraz, Iran; ^4^Department of Persian Medicine, School of Persian Medicine, Shahid Sadoughi University of Medical Sciences, Ardakan, Yazd, Iran; ^5^Center for Integrative Medicine and Planetary Health, University Hospital Essen, University of Duisburg-Essen, Essen, Germany; ^6^Department of Traditional Pharmacy and Persian Medicine, Faculty of Pharmacy and Pharmaceutical Sciences, Tehran Medical Sciences, Islamic Azad University, Tehran, Iran

**Keywords:** curcumin, inflammatory bowel disease, Crohn disease, ulcerative colitis, integrative medicine, herbal medicine

## Abstract

**Introduction:**

Inflammatory Bowel Disease (IBD), encompassing Crohn disease (CD) and ulcerative colitis (UC), presents complex challenges in management due to dysregulated immune responses and genetic predispositions. This study explored the potential of curcumin as an adjunctive therapy in IBD, assessing its efficacy and safety through a systematic review of clinical trials to enhance treatment strategies and outcomes.

**Methods:**

To identify placebo-controlled randomized clinical trials on curcumin treatment in IBD, databases such as Medline/PubMed, Scopus, Embase, Web of Knowledge, and Google Scholar were searched till May 2024. Inclusion criteria focused on RCTs comparing curcumin with placebo in IBD patients, with data extraction and analysis conducted using established methodologies and tools for comprehensive synthesis and assessment of study findings.

**Results:**

In this meta-analysis, 13 placebo-controlled RCTs on curcumin treatment in IBD were included after screening 362 records and conducting a full-text review. Most trials focused on UC patients and were published post-2010, utilizing oral curcumin with varying dosages and durations. The analysis showed curcumin’s significant efficacy in achieving clinical remission and response in UC patients, with heterogeneity observed. Adverse events and withdrawal rates did not significantly differ between curcumin and placebo groups. In CD patients, curcumin did not show superiority over placebo for clinical and endoscopic remission.

**Conclusion:**

The findings highlight curcumin’s potential as a treatment for UC but indicate inconclusive results for CD, emphasizing the need for further research. The multifaceted mechanisms of curcumin’s efficacy in IBD involve anti-inflammatory, antioxidant, microbiota modulatory, and immune-regulating properties. Further research is warranted to enhance understanding and treatment efficacy.

**Systematic review registration:**

https://www.crd.york.ac.uk/PROSPERO/view/CRD42024567247.

## 1 Introduction

Inflammatory Bowel Disease (IBD), including Crohn’s disease (CD) and ulcerative colitis (UC), are chronic, relapsing inflammatory conditions of the gastrointestinal tract that significantly impact the quality of life of affected individuals (1). The complex pathogenesis of IBD involves dysregulated immune responses, genetic predisposition, and environmental factors, making their management challenging (2). Routine treatment strategies often aim to suppress inflammation and manage symptoms, but they may be associated with adverse effects and limited efficacy in achieving long-term remission (3). Long-term use of immunosuppressant medications increases infection and malignancy risks, too (4). Exploring herbal medicine as an alternative approach is gaining traction due to its multifaceted pharmacological benefits, safety advantages, and holistic health approach (5, 6).

The geographical distribution of curcumin production has expanded to various parts of the world, ranging from Southern Asia to Central and South America. Curcumin (*Curcuma longa* L.), a polyphenolic compound derived from the turmeric plant, has gained attention in recent years for its anti-inflammatory, antioxidant, and immune-modulatory properties (7). Preclinical studies and observational data suggest that curcumin could have a therapeutic role in IBD by targeting key inflammatory pathways and promoting mucosal healing (8). However, the clinical efficacy of curcumin in the treatment of IBD remains a topic of debate, necessitating a comprehensive evaluation of the available evidence.

By critically assessing the efficacy and safety of curcumin supplementation in IBD patients, this study sought to provide valuable insights into the potential role of curcumin as an adjunctive therapy in the management of IBD. The findings of this review may inform clinical practice, guide future research directions, and contribute to optimizing treatment strategies for individuals living with IBD. This evidence-based approach will help clarify the therapeutic potential of curcumin and broaden our understanding of its role in the clinical management of IBD. This systematic review and meta-analysis aimed to synthesize the existing literature on the clinical use of curcumin for the treatment of IBD, focusing on placebo-controlled randomized clinical trials.

## 2 Methods

### 2.1 Search strategy and databases

The protocol for this study is registered in PROSPERO (CRD42024567247). To identify placebo-controlled randomized clinical trials evaluating the clinical outcomes of curcumin treatment in IBD patients, we searched Medline/PubMed, Scopus, Embase, Web of Knowledge, Cochrane, and Google Scholar databases. The searches were conducted using relevant queries for “title and abstract” up to May 2024, with restrictions to English-language publications (Supplementary Table 1).

### 2.2 Inclusion/exclusion criteria

We included randomized controlled trials (RCTs) that compared curcumin with placebo in patients with any form of IBD, regardless of the type of curcumin preparation, route of administration, concomitant treatments, disease severity, and criteria for outcome measures. The authors also performed a snowball search through the references of the chosen manuscripts to include additional articles that were not part of this study. Studies were excluded if: (1) outcome measures for clinical effectiveness (i.e., clinical remission, endoscopic remission, and clinical response) were not clearly defined or could not be extracted; (2) the design was non-randomized, single-arm trials, observational studies, or case-series; (3) the full text was not available; (4) the reported result was repetitive data from another RCT; or (5) the study was not written in English.

### 2.3 Study selection

The articles identified were managed using Mendeley. Four independent researchers (SM, HG, NP, MS) independently screened the aggregated library obtained from the database searches, read the full texts to evaluate eligibility (second screening), extracted or transformed the target data from the eligible studies, and assessed the risk of bias (RoB) for the studies included in the quantitative analysis. Any disagreements during these processes were resolved through discussion, achieving consensus in all cases.

### 2.4 Data extraction

A comprehensive spreadsheet was prepared to include all targeted information and statistics from the studies included in the quantitative analysis. This checklist comprised: general data (first author’s name, publication year, study period, and country), intervention descriptions (intervention and comparator, daily dose, intervention duration, daily use interval, and concomitant treatment), group-specific data (study design, setting, IBD characteristics [type, severity, anatomical location], intention-to-treat (ITT) and per-protocol (PP) sample sizes, mean age, male-to-female ratio), outcomes (clinical remission, endoscopic remission, clinical response, drug-related adverse events, and withdrawal rates) with their definitions, and the Cochrane Collaboration’s tool for assessing RoB. Outcomes were extracted using both ITT and PP methods. The quality assessment used the Cochrane Collaboration tool, evaluating each study on (1) random sequence generation, (2) allocation concealment, (3) blinding of participants and personnel, (4) blinding of outcome assessments, (5) incomplete outcome data, and (6) selective reporting. Each study was ranked as having a “low risk of bias,” “high risk of bias,” or “unclear” for these items.

### 2.5 Statistical analysis

Statistical analysis was conducted using RevMan software (Cochrane Collaboration; v5.4.1; released Sep. 2020). As all included study outcomes were dichotomous, the pooled estimates (summary or overall effect sizes) were reported as risk ratios (RR) with 95% confidence intervals (CI). Forest plots were constructed to visualize the effect sizes (with 95% CIs) of individual studies and the calculated summary effect size (with 95% CI). Heterogeneity around the summary effect size was assessed using the *χ*^2^ test and *I*^2^ statistic, with *p*-values <0.1 or an *I*^2^ > 50% indicating significant heterogeneity. Additionally, the Z(u) test was used for hypothesis testing of group comparisons, with a *p*-value <0.05 indicating statistical significance. Due to high heterogeneity, random-effects models were used to calculate the summary effect size.

### 2.6 Ethical considerations

No ethical approval was deemed necessary for the systematic review and meta-analysis, as the data were obtained from already published sources.

## 3 Results

### 3.1 Description of included studies into the meta-analysis

**Figure 1:**
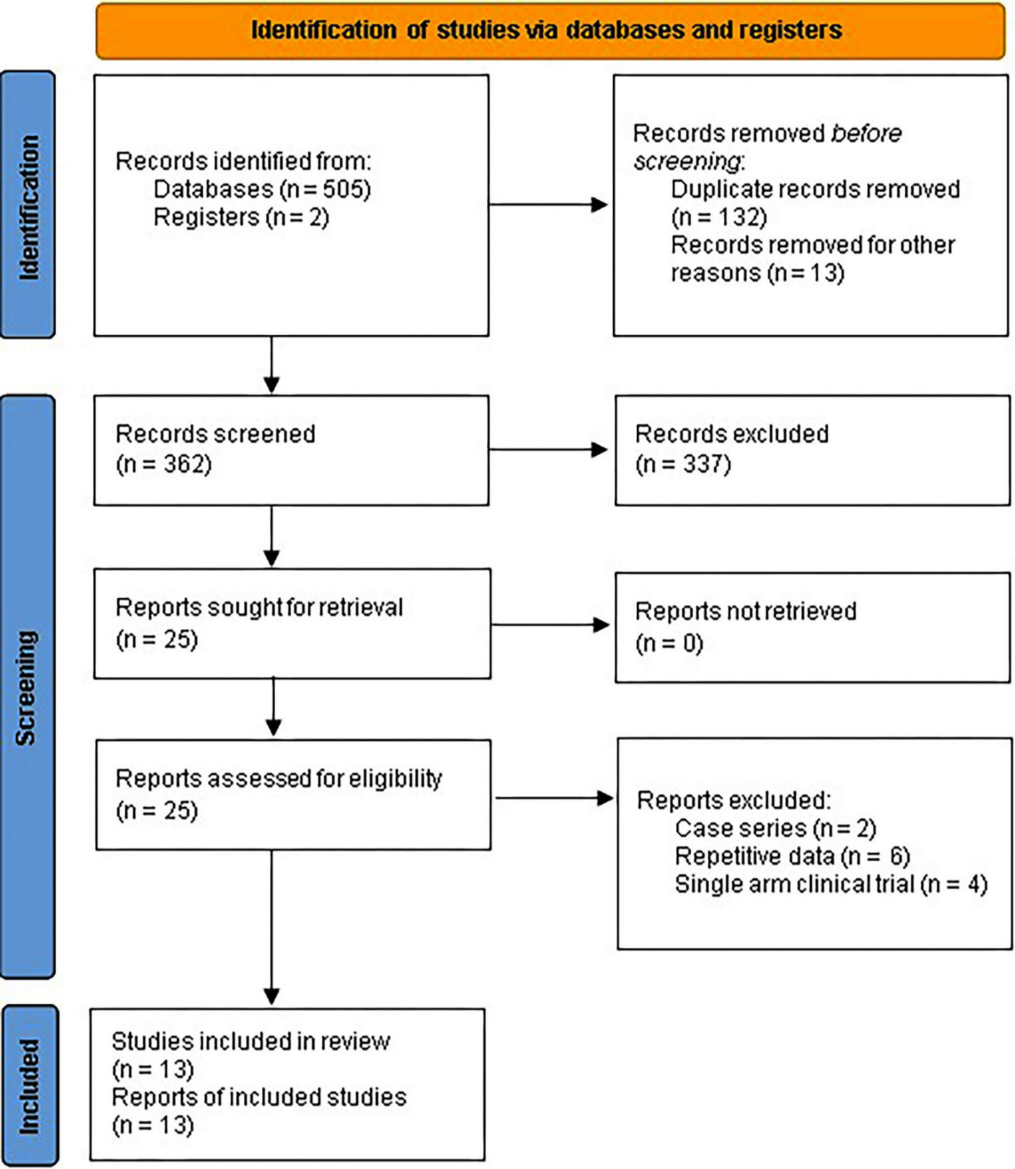
PRISMA 2020 flow diagram for systematic review and meta-analysis of placebo-controlled RCTs assessing curcumin efficacy for IBD.

Initially, 507 records were retrieved through a systematic search of databases. After removing duplicates, 362 records underwent screening for retrieval, resulting in 25 records. These records were assessed for eligibility through a full-text review. Finally, after excluding 12 records, 13 placebo-controlled RCTs were included in the meta-analysis (9–21) (Figure 1).

Eleven RCTs were conducted among UC patients and two among CD patients (20, 21). Nearly all trials were published after 2010. The aggregated sample sizes designated to receive curcumin and placebo were 322 and 308 in RCTs involving UC patients, and 51 and 41 in RCTs involving CD patients, respectively. The total randomized sample sizes in the included RCTs ranged from 30 to 89.

Of these 13 RCTs, all utilized oral purified curcumin as the intervention, except for 5 RCTs that utilized dual-drug combinations such as Curcumin-QingDai (CurQD) (10), curcumin enema (NCB-02) (13), curcuminoids nanomicelles (14), bioenhanced curcumin (BEC; VALDONE) (19), and curcumin derivative (Theracurmin^®^) (20). Seven RCTs were conducted among patients with mild-to-moderate UC.

**Figure 2:**
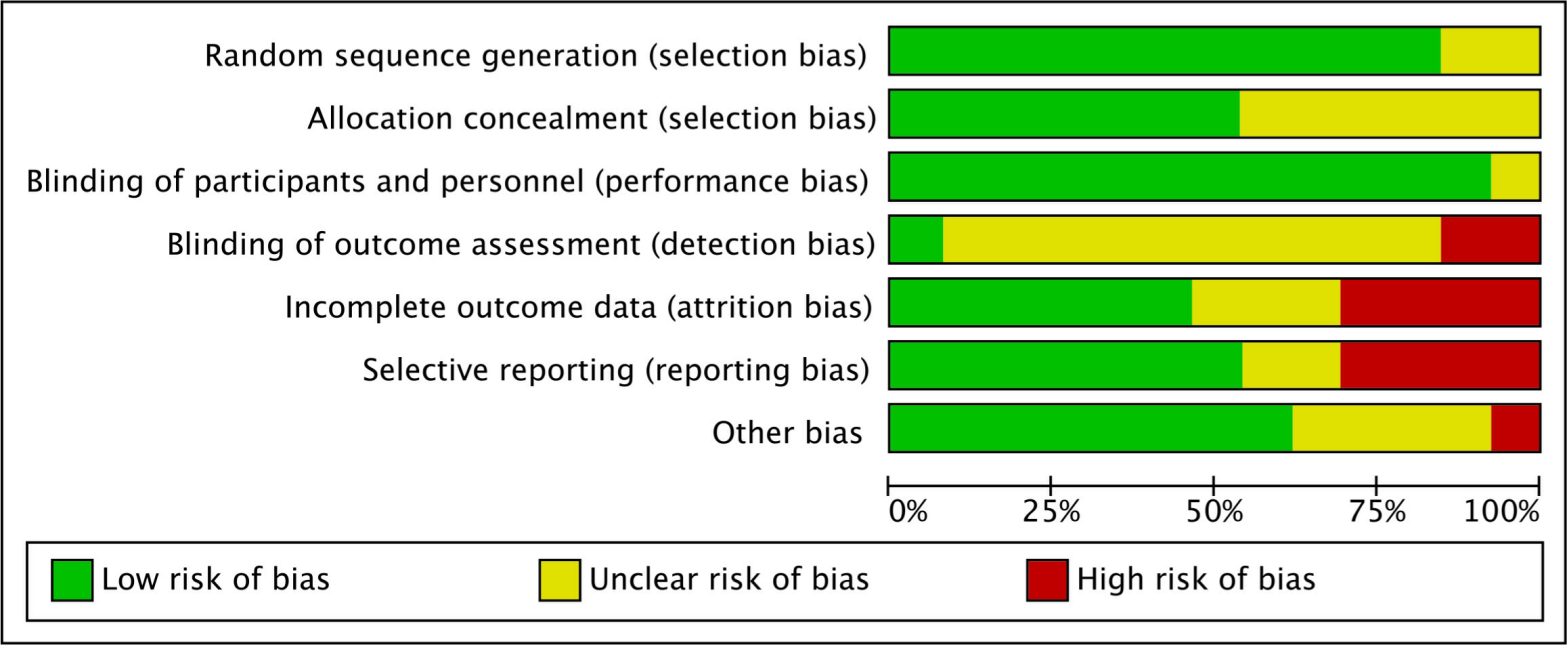
ROB graph of 13 included RCTs in the meta-analysis.

Furthermore, the cumulative daily dose of curcumin ranged from 0.1 to 10 g, with treatment durations ranging from 1 to 6 months. The most common concomitant treatment was mesalamine. Tables 1, 2 provide the relevant data retrieved from the included RCTs involving UC and CD patients, respectively. A summary of the Risk of Bias (RoB) in these studies is depicted in Figure 2 and Supplementary Figure 1.

**Table 1:** Retrieved data from 11 placebo-controlled RCT investigating the clinical efficacy of curcumin in UC.

Author	Kedia et al. (9)	Ben-Horin et al. (10)	Hanai et al. (11)	Lang et al. (12)	Singla et al. (13)	Masoodi et al. (14)	Banerjee et al. (15)	Kumar et al. (16)	Sadeghi et al. (17)	Shivakumar et al. (18)	Banerjee et al. (19)
Study type	Double-blind RCT	Multicenter 2-phase double-blind RCT [part II included]	Multicenter double-blind RCT	Multicenter double-blind RCT	Double-blind pilot RCT	Double-blind RCT	Double-blind RCT	Double-blind RCT	Double-blind RCT	RCT	Double-blind pilot RCT
Publication year	2017	2024	2006	2015	2014	2018	2017	2019	2020	2011	2021
Study period	2003–5	N/A	2004–5	2011–14	N/A	2017	2016–17	N/A	2018	N/A	2016–17
Country	India	Israel/Greece	Japan	Israel/Hong Kong/Cyprus	India	Iran	India	India	Iran	India	India
Patients											
Severity	Mild-to-moderate	Mild-to-severe UC	Quiescent (inactive)	Mild-to-moderate	Mild-to-moderate UC	Mild-to-moderate	Mild-to-moderate	Mild-to-severe UC	Mild-to-moderate	Active UC	Mild-to-moderate
Anatomical location	Proctitis/left colitis/pancolitis	Left colitis/extensive	–	Proctitis/left colitis/extensive	Proctitis/proctosigmoiditis	Proctitis/left colitis/extensive	–	–	Proctitis/left colitis/extensive/pancolitis	–	Left colitis/pancolitis
Intervention	Curcumin	Curcumin-QingDai (CurQD)	Curcumin	Curcumin	Curcumin enema [NCB-02]	Curcuminoids nanomicelles	SMEDDS Curcumin	*Curcuma longa*	Curcumin	Curcumin	Bioenhanced Curcumin (BEC; VALDONE)
Mean age	36 ± 12	35 [IQR: 23–48]	45.2 ± 15.8	40.4 ± 12.7	32.7 ± 8.9	38.2 ± 16.4	N/A	N/A	40.1 ± 13.2	N/A	38 [median]
M/F	1.23	1.15	1.05	1.09	1.09	1.15	N/A	N/A	0.30	N/A	1.83
Sample size											
ITT	29	28	45	26	23	28	22	28	35	–	34
PP	16	23	43	25	14	28	19	–	31	24	30
Daily dose	150 mg TID	500 mg + 500 mg TID	1 g BID	1.5 g BID	140 mg (enema)	80 mg TID	50 mg QD/BID	10 g N/A	500 mg TID	10 g N/A	50 mg BID
Comparator	Placebo	Placebo	Placebo	Placebo	Placebo	Placebo	Placebo	Placebo	Placebo	Placebo	Placebo
Mean age	34 ± 7	25 [IQR: 23–30]	39.7 ± 14.2	41.4 ± 13.9	35.5 ± 13.8	36.0 ± 11.8	N/A	N/A	40.6 ± 12.4	N/A	38 [median]
M/F	3.12	0.625	1.44	2.00	1.00	0.87	N/A	N/A	0.59	N/A	1.50
Sample size											
ITT	33	13	44	24	22	29	25	25	35	–	35
PP	25	7	39	22	16	28	23	–	32	23	32
Intervention duration	2 months	2 months	6 months	1 month	2 months	1 month	3 months	2 months	2 months	2 months	1.5 months
Concomitant treatment	ML [2.4 g QD]	SOC	SZ [1–3 g QD] / ML [1.5–3 g QD]	ML [4 g QD]	ML [0.8 g BID]	ML [3 g QD]	ML	ML [2.4 g QD]	ML	ML	ML [1 g QD]
Outcomes											
*Clinical remission*											
Definition	DAI ≤2^e^	DAI ≤ 2	DAI ≤ 4	DAI ≤ 2	DAI ≤ 3	–^d^	–	–	DAI ≤ 2	–	PMS ≤ 1
Intervention											
ITT	9	14	43	14	10				26		15
PP	9	–	41	15	10				–		15
Comparator											
ITT	9	1	36	0	5				14		0
PP	9	–	31	0	5				–		0
*Endoscopic remission*^a^											
Definition	BES ≤ 1	MES ≤ 1	–	MES ≤ 1	↓MES ≥ 1^a^		MES ≤ 1	–		↓MES ≥1^a^	MES ≤ 1
Intervention											
ITT	10	21		–^c^	–		5			–^b^	14
PP	10	21		8^b^	12		0			15	14
Comparator											
ITT	10	3		–^c^	–		5			–^b^	0
PP	10	3		0^b^	8		0			10	0
*Clinical response*											
Definition	↓DAI ≥ 3	↓DAI ≥ 3	–	↓DAI ≥3	↓DAI ≥ 3	–	↓PMS ≥ 3	↓DAI ≥ 3^c^	↓DAI ≥ 3	–	↓PMS ≥ 2
Intervention											
ITT	6	24		17	13		12	17	–		18
PP	6	–		17	13		12	–	29		18
Comparator											
ITT	12	4		3	8		5	13	–		5
PP	12	–		3	8		5	–	19		5
*AEs*											
Intervention	0	1	7	–	0	5	–	8	1	–	1
Comparator	1	0	N/A		0	3		8	0		2
Withdrawal rate											
Intervention	13	5	2	1	9	0	3	–	4	–	4
Comparator	8	6	5	2	6	1	2		3		3

a Studies with endoscopic improvement as an outcome are also included.

b Out of 22 and 16 subjects in intervention and comparator groups, respectively.

c Mean change in MES between intervention and comparator groups: −0.55 ± 0.79 vs. 0.15 ± 0.49.

d Endpoint SCCAI comparison between intervention and comparator groups: 1.71 ± 1.84 vs. 2.68 ± 2.09.

e CAI/SCCAI/UCDA.

ML, mesalamine; SZ, sulfasalazine; DAI, disease activity index; MES, partial Mayo endoscopic score; BES, Baron endoscopic score; PMS, partial Mayo score; AEs, adverse events; RCT, randomized controlled clinical trial; UC, ulcerative colitis; ITT, intention-to-treat; PP, per-protocol; M/F, male-to-female ratio.

**Table 2:** Retrieved data from 2 placebo-controlled RCT investigating the clinical efficacy of curcumin in CD.

Author	Sugimoto et al. (20)	Bommelaer et al. (21)
Study type	Multicenter double-blind RCT	Multicenter double-blind RCT
Publication year	2020	2019
Study period	2015–17	2014–18
Country	Japan	France
Patients		
Severity	Mild-to-moderate active CD	CD with bowel resection
Anatomical location	[ileal/colonic/ileo-colonic]	ileal/colonic/ileo-colonic
Intervention	Theracurmin® [Curcumin derivative]	Curcumin
Mean age	36.3 ± 8.9	35.0 ± 10.5
M/F	1.86	0.94
Sample size		
ITT	20	31
PP	17	26
Daily dose	180 mg BID	1 g TID
Comparator	Placebo	Placebo
Mean age	32.9 ± 13.4	37.6 ± 13.8
M/F	4.00	0.24
Sample size		
ITT	10	31
PP	9	27
Intervention duration	3 months	6 months
Concomitant treatment	SOC	AZA [2–2.5 mg/kg QD]
Outcomes		
*Clinical remission*		
Definition	CDAI <150	CDAI<150
Intervention		
ITT	8	19
PP	–	19^a^
Comparator		
ITT	0	17
PP	–	17^a^
*Endoscopic remission*		
Definition	SESCD ≤4	Rutgeerts’ index score < i2
Intervention		
ITT	3	10
PP	3	10
Comparator		
ITT	0	13
PP	0	13
*AEs*		
Intervention	0	5
Comparator	0	2
Withdrawal rate		
Intervention	3	5
Comparator	1	4

a Out of 23 subjects in both groups.

AZA, Azathioprine; SOC, standard of care; CDAI, CD activity index; SESCD, simple endoscopic score for Crohn’s disease; AEs, adverse events; RCT, randomized controlled clinical trial; CD, Crohn disease; ITT, intention-to-treat; PP, per-protocol; M/F, male-to-female ratio.

### 3.2 Clinical efficacy of curcumin versus placebo in UC

**Figure 3:**
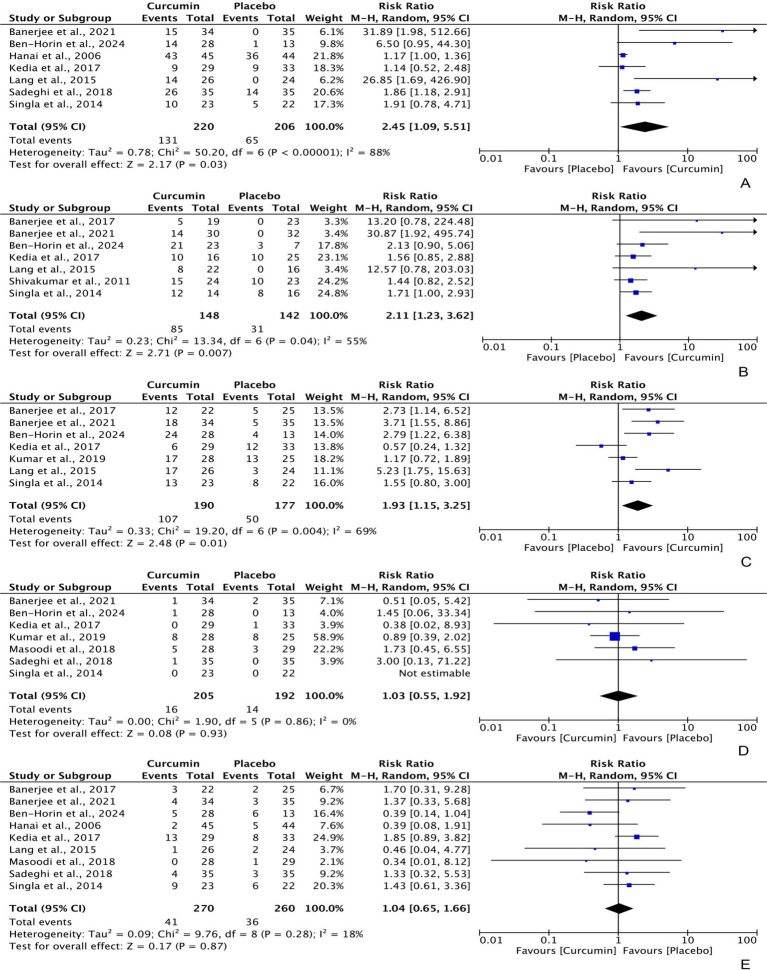
Forest plots of comparing curcumin with placebo in improving UC in terms of **(A)** clinical remission [PP] = RR: 3.04 [95% CI: 0.93, 9.96], Z: 1.84, P: 0.07; **(B)** endoscopic remission [ITT] = RR: 3.81 [95% CI: 0.95, 15.18], Z: 1.89, P: 0.06; **(C)** clinical response [PP] = RR: 2.04 [95% CI: 1.30, 3.20], Z: 3.08, P: 0.002.

Intention-to-treat data regarding the efficacy of curcumin for achieving clinical remission and clinical response in UC patients were extracted from 7 different RCTs, with 220 and 190 participants in the curcumin group, and 206 and 177 participants in the placebo group, respectively. The analysis produced a combined relative risk (RR) of 2.45 (95% CI: 1.09, 5.51; Z: 2.17, P: 0.03) for clinical remission (Figure 3A) and 1.93 (95% CI: 1.15, 3.25; Z: 2.48, P: 0.01) for clinical response (Figure 3C), both of which favored curcumin significantly. Notably, significant heterogeneity was observed in both analyses (clinical remission = χ^2^: 50.20, *p* < 0.0001, I^2^: 88%; clinical response = χ^2^: 19.20, P: 0.004, I^2^: 69%).

Furthermore, the per-protocol analyses also showed a significant difference in clinical response, with an RR of 2.04 (95% CI: 1.30, 3.20; Z: 3.08, P: 0.002); however, the difference between curcumin and placebo was marginally insignificant for clinical remission, with an RR of 3.04 (95% CI: 0.93, 9.96; Z: 1.84, P: 0.07).

Based on per-protocol data from 7 RCTs (148 participants in the curcumin group and 142 participants in the placebo group), curcumin demonstrated statistically higher efficacy in achieving UC endoscopic remission compared to placebo, with an RR of 2.11 (95% CI: 1.23, 3.62; Z: 2.71, P: 0.007). There was moderate heterogeneity observed among these RCTs (χ^2^: 0.23, P: 0.04, I^2^: 55%) (Figure 3B). However, the intention-to-treat data did not show a significant result, with an RR of 3.81 (95% CI: 0.95, 15.18; Z: 1.89, P: 0.06).

Among the participants, 16 (7.80%) experienced adverse events (AEs) and 41 (15.16%) withdrew from the intervention in the curcumin group, compared to 14 (7.29%) and 36 (13.85%) of participants in the placebo group. The pooled analysis indicated no significant difference in the frequency of AEs, with an RR of 1.03 (95% CI: 0.55, 1.92; Z: 0.08, P: 0.93) (Figure 3D), and withdrawal rates, with an RR of 1.04 (95% CI: 0.65, 1.66; Z: 0.17, P: 0.87) (Figure 3E), between the curcumin and placebo groups, although lower rates were reported in the placebo group. There was no heterogeneity detected in these studies (AEs frequency = χ^2^: 1.90, P: 0.86, I^2^: 0%; withdrawal rates = χ^2^: 9.76, P: 0.28, I^2^: 18%).

### 3.3 Clinical efficacy of curcumin versus placebo in CD

**Figure 4:**
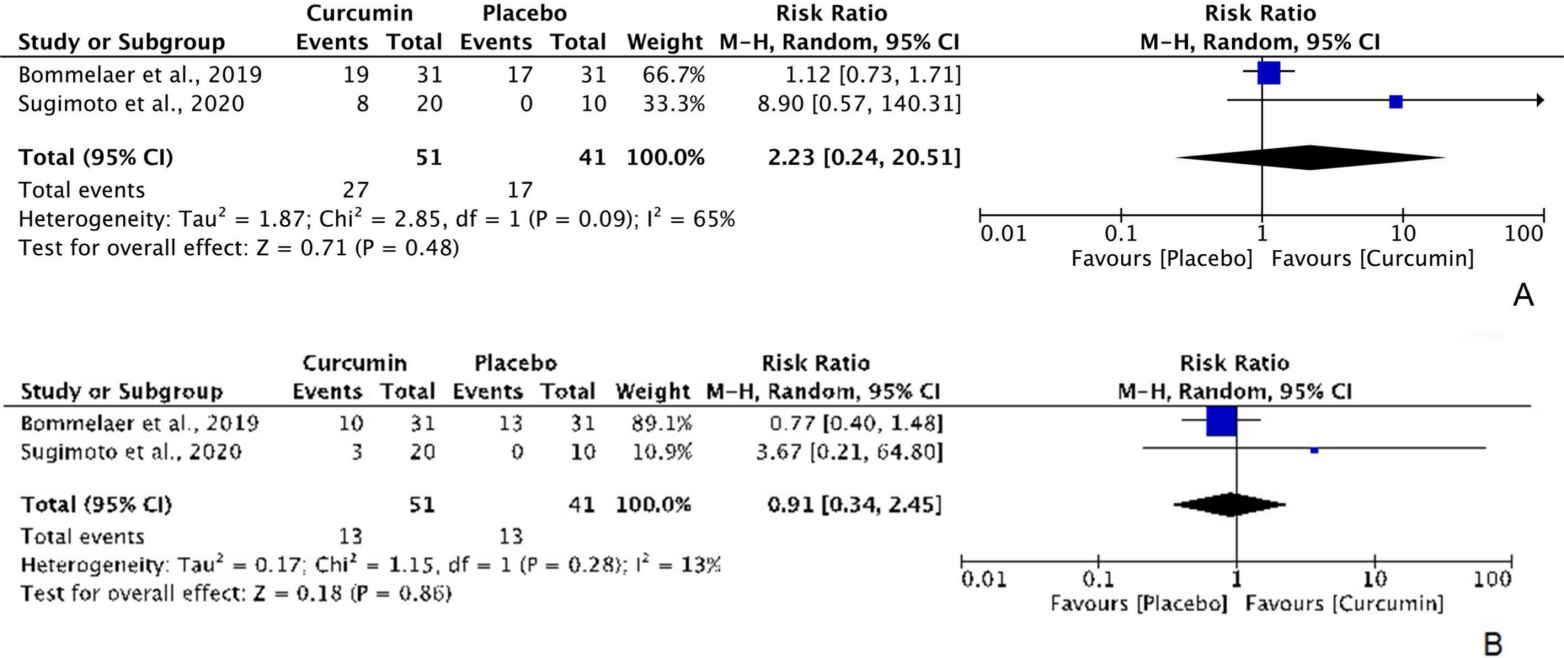
Forest plots of comparing curcumin with placebo in improving CD in terms of **(A)** clinical remission [ITT] and **(B)** endoscopic remission [ITT].

According to the intention-to-treat data from 2 RCTs evaluating the efficacy of curcumin for clinical and endoscopic remission in CD patients (with 51 participants in the curcumin group and 41 participants in the placebo group), curcumin did not show significant superiority over placebo in terms of clinical remission, with an RR of 2.23 (95% CI: 0.24, 20.51; Z: 0.71, P: 0.48; χ^2^: 2.85, P: 0.09, I^2^: 65%) (Figure 4A) and endoscopic remission, with an RR of 0.91 (95% CI: 0.34, 2.45; Z: 0.18, P: 0.86; χ^2^: 1.15, P: 0.28, I^2^: 13%) (Figure 4B).

## 4 Discussion

This meta-analysis of 13 placebo-controlled RCTs found that curcumin showed significant benefits in achieving remission and clinical response in UC patients. However, in CD patients, curcumin did not show superiority over placebo for remission or endoscopic improvements.

The efficacy of curcumin for IBD is believed to be attributed to its various mechanisms of actions. Curcumin has potent anti-inflammatory effects by inhibiting inflammatory pathways such as NF-κB (nuclear factor kappa-light-chain-enhancer of activated B cells) and cytokines involved in the inflammatory response (22). Curcumin acts as an antioxidant, scavenging free radicals, and reducing oxidative stress, which is known to play a role in the pathogenesis of IBD (2, 23). Curcumin has been shown to influence the composition of the gut microbiota, promoting a healthy balance of beneficial bacteria, which can help in the management of IBD (24). It can modulate the immune system by regulating immune cell function, cytokine production, and immune reactions, thereby potentially reducing excessive inflammation seen in IBD (25). Curcumin can also help maintain the integrity of the intestinal barrier by enhancing the expression of tight junction proteins, which may prevent leakage of harmful substances into the bloodstream and reduce inflammation in the gut (26). Curcumin can inhibit enzymes such as cyclooxygenase-2 (COX-2) and lipoxygenase (LOX) that are involved in the inflammatory pathways in IBD, too (27). These mechanisms collectively contribute to the potential efficacy of curcumin in managing IBD.

Curcumin is used in different parts of the world for managing IBD, such as ulcerative colitis and Crohn’s disease. It is commonly consumed as a dietary supplement or included in traditional remedies in various cultures. Research on the use of curcumin for treating IBD has been conducted in various countries around the world such as: United States, India, United Kingdom, Australia, Canada, China, Germany, South Korea, Iran, and Italy published in scientific journals, clinical trials, and reviews exploring the efficacy, mechanisms of action, and safety of curcumin in the context of IBD (28–30).

The failure rate of routine medical systems to treat IBD can vary among individuals. It is important to note that conventional treatments for IBD, including medications such as anti-inflammatory drugs, immunosuppressants, biologics, and surgery, are generally effective in managing symptoms and inducing remission in many patients (3). However, some individuals may experience treatment failures for various reasons, including: (1) disease severity; in some cases, IBD may be more severe or aggressive, making it challenging to achieve adequate symptom control and disease remission with standard treatments. (2) Individual differences; each person’s response to treatment can vary based on factors such as genetics, immune system function, and presence of comorbidities, which can influence the effectiveness of medications. (3) Development of drug resistance; over time, some individuals with IBD may develop resistance to certain medications, leading to treatment failure and the need to explore alternative therapies. (4) Adverse reactions; side effects or intolerances to medications used to treat IBD can result in treatment discontinuation or failure to achieve desired outcomes. (5) Non-adherence to treatment; failure to adhere to prescribed medication regimens, lifestyle modifications, or follow-up appointments can also contribute to treatment failure in managing IBD (3, 31). While the failure rate of routine medical systems in treating IBD is not specifically quantified, healthcare providers work closely with patients to monitor disease activity, adjust treatment plans as needed, and explore alternative therapies to improve outcomes for individuals with IBD (4, 32). A multidisciplinary approach including use of evidence-based herbal preparations to care can help enhance the effectiveness of treatment strategies for IBD.

While curcumin is generally considered safe for most people when taken in appropriate doses, there are some potential side effects and considerations to be aware of when using curcumin for treating IBD. Some possible side effects of curcumin supplementation include: (1) Gastrointestinal issues; high doses of curcumin may cause gastrointestinal discomfort, such as nausea, bloating, and diarrhea, particularly in individuals with sensitive stomachs or digestive issues (33). (2) Interaction with medications; curcumin may interact with certain medications, such as beta blockers, blood thinners, antiplatelet drugs, and medications that affect blood sugar levels (34–36). (3) Allergic reactions; some individuals may be allergic to curcumin or components of turmeric, leading to allergic reactions such as skin rash, itching, or swelling (37). (4) Blood clotting; curcumin may have antithrombotic properties, which could increase the risk of bleeding in individuals with bleeding disorders or those taking anticoagulant medications (38). (5) Pregnancy and breastfeeding; pregnant and breastfeeding women should consult with a healthcare provider before using curcumin supplements, as its safety during pregnancy and lactation is not well-established (39). (6) Iron absorption; curcumin may inhibit iron absorption in the body, which could be a concern for individuals with iron deficiency anemia or those at risk of iron deficiency (40). Therefore, monitoring for adverse reactions and discussing any changes in symptoms or health status while using curcumin is also advisable.

UC is primarily characterized by continuous inflammation of the colon’s mucosal layer, often associated with superficial ulceration. In contrast, CD can affect any part of the gastrointestinal tract and manifests with transmural inflammation (3). This fundamental difference in pathology may influence the efficacy of curcumin, given its predominantly anti-inflammatory properties, which may be more advantageous in a predominantly mucosal disease like UC.

Furthermore, we will address sample size considerations by acknowledging that our cohort’s composition may limit the generalizability of our findings. Smaller sample sizes can lead to variability in response rates and may not adequately represent the broader patient population. We will suggest that future studies with larger and more diverse cohorts are warranted to further elucidate the therapeutic roles of curcumin in both UC and CD. Finally, the variations in current treatment protocols for UC and CD (1) may also play a significant role in how effective curcumin is perceived across these conditions.

Curcumin has demonstrated promising effects in managing rheumatoid arthritis (RA), as evidenced by a study encompassing six publications with a total of 539 patients (41). The activity of RA was assessed using various clinical measures, including erythrocyte sedimentation rate (ESR), disease activity score (DAS), tender joint count (TJC), and swollen joint count (SJC). Notably, significant improvements were observed in ESR (MD = −29.47, *p* = 0.02), DAS28 (MD = −1.20, *p* = 0.0003), SJC (MD = −5.33, p = 0.02), and TJC (MD = −6.33, *p* = 0.006) in patients treated with curcumin compared to controls. These findings highlight curcumin’s potential as an effective anti-inflammatory agent in reducing disease activity and improving patient outcomes in other inflammatory diseases, such as RA. Although there is currently no supporting evidence, additional research is necessary to thoroughly clarify the specific regulatory genes and pathways that curcumin targets in these diseases.

We recognize that racial and ethnic factors can significantly impact health outcomes, disease prevalence, and treatment responses in IBD patients (42). While there is evidence indicating a higher risk of IBD among individuals of white ethnicity, we could not find sufficient evidence in our included trials to support this point in the current study (43) due to a lack of information on patients’ ethnicity. It is important to consider this factor in future trials involving IBD patients to explore any possible correlation between race and treatment response.

The present systematic review and meta-analysis study faces certain constraints, such as a notable proportion of low-quality pooled studies, research conducted in settings of varying quality, different curcumin dosage forms, and samples that might not accurately represent the broader community. Nonetheless, to overcome these limitations, it is essential to prioritize specific actions in ongoing research, such as incorporating recently published trials. By addressing these issues, this systematic review and meta-analysis study has the potential to enhance the credibility of its results and advance our comprehension of the efficacy of curcumin in alleviating IBD like UC and CD.

## 5 Conclusion

In general, the findings indicate that curcumin might be more beneficial in addressing UC in contrast to CD, likely due to varying mechanisms like anti-inflammatory and antioxidant effects. Despite curcumin’s widespread use for IBD treatment worldwide, it’s crucial to take into account possible adverse reactions and interactions with medications.

To enhance future clinical trials, we recommend ensuring diverse patient recruitment to assess variations in treatment response, standardizing dosages and formulations of curcumin, and employing longitudinal designs to monitor long-term effects. It’s crucial to control for confounding variables like disease severity and include health-related quality of life assessments to capture the treatment’s overall impact. Additionally, incorporating mechanistic studies can help clarify the biological pathways through which curcumin operates. By adhering to these guidelines, future research can provide robust evidence regarding the efficacy and safety of curcumin in IBD, ultimately improving patient outcomes.

## Data Availability

The original contributions presented in the study are included in the article/Supplementary material, further inquiries can be directed to the corresponding author.
