# Supplementary material for: Curcumin for the clinical treatment of inflammatory bowel diseases: a systematic review and meta-analysis of placebo-controlled randomized clinical trials
Source: Front Nutr. 2025 Mar 24;12:1494351. doi: 10.3389/fnut.2025.1494351 (PMC11973083; doi:10.3389/fnut.2025.1494351)
Supplement: Supplementary file 1 [file Table_1.doc]

| Supplementary Table 1- Search strategy and obtained records till May 2024 | |
| --- | --- |
| **Database Name** | **Query** |
| **PubMed**  *(n = 64)* | ((((((((((curcumin[Title/Abstract]) OR ("curcuma domestica"[Title/Abstract])) OR ("curcuma longa"[Title/Abstract])) OR (turmeric[Title/Abstract])) OR (tumeric[Title/Abstract])) OR (curcuminoid*[Title/Abstract])) OR ("NCB-02"[Title/Abstract])) OR (Curcumin[MeSH Terms])) AND (((((("inflammatory bowel disease*"[Title/Abstract]) OR ("crohn*"[Title/Abstract])) OR ("ulcerative colitis"[Title/Abstract])) OR ("colitis, ulcerative"[MeSH Terms])) OR ("Crohn Disease"[MeSH Terms])) OR ("Inflammatory Bowel Diseases"[MeSH Terms]))) AND (((((((("Randomized Controlled Trial"[Publication Type]) OR ("Controlled Clinical Trial"[Publication Type])) OR ("Clinical Trial"[Publication Type])) OR ("Clinical Trials as Topic")) OR (randomized[Title/Abstract])) OR (placebo[Title/Abstract])) OR (random*[Title/Abstract])) OR (trial[Title/Abstract]))) NOT ((animals[MeSH Terms]) NOT (humans[MeSH Terms])) |
| **Scopus**  *(n = 332)* | (TITLE-ABS-KEY(curcumin) OR TITLE-ABS-KEY("curcuma domestica") OR TITLE-ABS-KEY("curcuma longa") OR TITLE-ABS-KEY(turmeric) OR TITLE-ABS-KEY(tumeric) OR TITLE-ABS-KEY(curcuminoid*) OR TITLE-ABS-KEY("NCB-02")) AND (TITLE-ABS-KEY("inflammatory bowel disease*") OR TITLE-ABS-KEY("crohn*") OR TITLE-ABS-KEY("ulcerative colitis")) AND (TITLE-ABS-KEY(random*) OR TITLE-ABS-KEY(randomized) OR TITLE-ABS-KEY(placebo) OR TITLE-ABS-KEY(trial) OR TITLE-ABS-KEY(RCT)) |
| **Embase**  *(n = 109)* | (curcumin:ab,ti OR 'curcuma domestica':ab,ti OR 'curcuma longa':ab,ti OR turmeric:ab,ti OR tumeric:ab,ti OR curcuminoid*:ab,ti OR 'NCB-02':ab,ti) AND ('inflammatory bowel disease*':ab,ti OR 'crohn*':ab,ti OR 'ulcerative colitis':ab,ti) AND (random*:ab,ti OR randomized:ab,ti OR placebo:ab,ti OR trial:ab,ti OR rct:ab,ti) |
